# Co-Occurrence of Stunting and Off-Track Early Child Development in Low- and Middle-Income Countries

**DOI:** 10.1001/jamanetworkopen.2024.62263

**Published:** 2025-03-04

**Authors:** Joshua Jeong, Hyejun Chi, Lilia Bliznashka, Helen O. Pitchik, Rockli Kim

**Affiliations:** 1 Hubert Department of Global Health, Rollins School of Public Health, Emory University, Atlanta, Georgia; 2 Interdisciplinary Program in Precision Public Health, Department of Public Health Sciences, Graduate School of Korea University, Seoul, Republic of Korea; 3 International Food Policy Research Institute, Washington, DC; 4 Global Academy of Agriculture and Food Systems, University of Edinburgh, Scotland, United Kingdom; 5 Division of Epidemiology, University of California Berkeley School of Public Health, Berkeley; 6 Division of Health Policy and Management, College of Health Sciences, Korea University, Seoul, Republic of Korea

## Abstract

**Importance:**

Although children across low- and middle-income countries (LMICs) are increasingly surviving, many are not fully thriving. Both stunting and off-track early child development (ECD) hinder children’s potential to thrive.

**Objectives:**

To estimate the global prevalence of the co-occurrence of stunting and off-track ECD and explore its association with nurturing care and sociodemographic factors.

**Design, Setting, and Participants:**

This cross-sectional study pooled data from the most recent Demographic and Health Surveys and Multiple Indicator Cluster Surveys (2010-2020) on 173 416 children aged 36-59 months in 41 LMICs. Statistical analysis was conducted from February to December 2024.

**Exposure:**

Risk factors pertaining to inadequate nurturing care, low socioeconomic status, and other sociodemographic characteristics.

**Main Outcomes and Measures:**

Children were classified into 4 groups with respect to thriving: children who were stunted only (height-for-age *z* score <−2 SD), off-track ECD only (measured using the Early Childhood Development Index), both stunted and off-track ECD (co-occurrence), or neither.

**Results:**

In the pooled sample of 173 416 children, the mean (SD) child age was 47.1 (6.8) months, and 88 242 (50.9%) were boys. Approximately 1 in 6 children (17.0% [95% CI, 16.8%-17.2%]) were both stunted and had off-track ECD, 17.1% (95% CI, 16.9%-17.3%) were stunted only, 27.8% (95% CI, 27.6%-28.0%) had off-track ECD only, and 38.1% (95% CI, 37.9%-38.4%) were neither stunted nor had off-track ECD. Socioeconomic gradients were observed, with more co-occurrence in lower-income countries (18.2% [95% CI, 17.9%-18.6%]), poorer households (22.1% [95% CI, 21.7%-22.5%] for poorest wealth quintile), mothers with lower educational levels (20.8% [95% CI, 20.6%-21.0%] for primary education or less), and rural settings (19.3% [95% CI, 19.1%-19.6%]). Various indicators of inadequate nurturing care along with low socioeconomic status were associated with co-occurrence. The top 5 factors associated with co-occurrence were poorest wealth quintile (adjusted odds ratio [AOR], 2.75; 95% CI, 2.53-2.99), no early childhood education (AOR, 2.22; 95% CI, 2.10-2.34), low maternal educational level (AOR, 1.44; 95% CI, 1.37-1.51), no toys at home (AOR, 1.43; 95% CI, 1.35-1.51), and diarrhea (AOR, 1.38; 95% CI, 1.31-1.45). The associations of poor household wealth, no birth registration, and no early childhood education with co-occurrence were significantly larger than their associations with stunting only or off-track ECD only.

**Conclusions and Relevance:**

This cross-sectional study of young children in LMICs suggests that a significant proportion were both stunted and had off-track ECD. These findings underscore the need for multisectoral interventions that holistically target nutrition, health, and ECD risks to ensure that all children globally can thrive, especially those facing the double burden of stunting and off-track ECD.

## Key Points

**Question:**

What is the prevalence of and what factors are associated with the co-occurrence of stunting and off-track early child development (ECD)?

**Findings:**

In this cross-sectional study of 173 416 children aged 3 to 4 years in 41 low- and middle-income countries, 1 in 6 children were both stunted and had off-track ECD. Multiple indicators of inadequate nurturing care along with low socioeconomic status were most associated with the co-occurrence of stunting and off-track ECD.

**Meaning:**

This study highlights the need for multisectoral interventions to support the most vulnerable group of children experiencing the double burden of stunting and off-track ECD and promote their full potential to thrive.

## Teaser

This cross-sectional study of children in low- and middle-income countries (LMICs) estimates the prevalence of the co-occurrence of stunting and off-track early child development and examines the factors associated with this co-occurrence.

## Introduction

In low- and middle-income countries (LMICs), millions of children are failing to reach their full developmental potential and capacity to thrive beginning from the earliest years of life.^1^ Thriving is conceptualized as a child’s capacity to achieve their full potential in terms of their health, development, and well-being.^2^ In order to thrive, children require multiple inputs, including adequate nutrition, health, security and safety, opportunities for responsive caregiving, and early learning, as well as an enabling environment, which are all core components recognized in the Nurturing Care Framework (NCF).^3^ The most commonly referenced population-level indicator for young children’s developmental potential is derived from national rates of stunting (ie, height-for-age *z* scores <−2 SD) and poverty.^4,5^ Using Demographic and Health Surveys (DHS) and Multiple Indicator Cluster Surveys (MICS) data available up to 2010, Lu et al^5^ estimated that 250 million children younger than 5 years (43%) were at risk of not fulfilling their developmental potential based on stunting and poverty.

Over the past decade, there has been increasing evidence clarifying the distinctions between stunting and potential neurodevelopmental delays or off-track early child development (ECD).^6,7,8^ Although stunting is a useful marker of cumulative early-life adversities and the inadequacy of the environment to which children have been exposed, it is not causally related to delayed child cognitive, motor, or socioemotional development.^9^ Maternal and child nutritional interventions aimed primarily at reducing stunting do not necessarily improve children’s neurodevelopment or longer-term functional outcomes.^6^ Thus, stunting on its own is an incomplete proxy for child thriving,^6^ and instead it is important to also monitor more direct child development indicators such as those who are off-track in their attainment of early milestones.

Global data on the status of off-track ECD have been propelled through the creation of new population-level measurement tools. The Early Child Development Index (ECDI) is currently the most mainstreamed global tool for ECD, which has been introduced within large-scale household surveys such as the DHS and MICS to provide a composite measure of early cognitive, physical, and socioemotional development milestones for children 36 to 59 months of age.^10^ The inclusion of the ECDI within DHS and MICS has facilitated cross-country comparisons and enabled global tracking for the Sustainable Development Goal (SDG) target 4.2.1 on ECD.^11^ The United Nations 2024 Progress Toward the SDGs Report estimated that approximately one-third of young children globally are developmentally off-track.^12,13^

Although stunting is not a cause of off-track ECD, they share common underlying risk factors that can impede children’s full potential to thrive.^2^ The NCF^14^ and UNICEF’s conceptual framework on child nutrition^15^ recognize how optimal nutrition and development are key components of thriving that are partly influenced by common underlying risks (eg, poverty, inadequate caregiving, infectious disease^6,16,17^). However, the prevalence of these outcomes has been mostly reported independently or even interchangeably, with some incorrectly using stunting as a proxy for off-track ECD.^9^ To our knowledge, the co-occurrence of stunting and off-track ECD has not been previously estimated at a population level. However, children experiencing the double burden of both stunting and off-track ECD are likely to face greater cumulative early-life adversities that further jeopardize their full potential to thrive compared with those who experience stunting or off-track ECD alone.

In this study, we assessed the global landscape of young children at risk of not thriving due to stunting and off-track ECD either independently or jointly. Our first objective was to estimate the proportion of children aged 36 to 59 months who were stunted only, had off-track ECD only, were both stunted and had off-track ECD, or neither were stunted nor had off-track ECD. Our second objective was to explore the individual-, household-, and community-level factors associated with the different categories of outcomes. Overall, this study aims to uncover a more comprehensive picture of young children who are not reaching their full potential to thrive, particularly regarding the subpopulation of those who are doubly burdened by both stunting and off-track ECD, which can inform strategies to support child thriving in LMICs.

## Methods

### Data Sources

In this cross-sectional study, we combined data from the DHS and MICS, which are nationally representative standardized household surveys that collect primarily health and nutrition indicators but also some ECD indicators in LMICs. Details on sampling procedures and data collection are reported elsewhere.^18,19^ Of the most recent publicly available survey for each LMIC (75 total: 29 DHS and 46 MICS), those conducted before 2009 (n = 9) were excluded because the ECDI had not yet been introduced.^10^ We also excluded surveys that did not assess both child’s height for age and ECD (n = 18) or any of the selected factors (n = 3). Surveys conducted between 2021 and 2022 were excluded (n = 4) because they contained a new measure for ECD that made it incompatible with the surveys from 2010 to 2020. Data from the child and household files in DHS and the woman and household files in MICS were merged to create the analytic sample of children aged 36 to 59 months (N = 195 691). After excluding those who were missing information for either height for age or ECD, the final analytic sample comprised 173 416 children from 41 LMICs (eTable 1 in Supplement 1). We used publicly available data with no identifiable information on survey participants. These activities do not meet the regulatory definition of human participants research. As such, institutional review board review and informed consent were not required. We followed the Strengthening the Reporting of Observational Studies in Epidemiology (STROBE) reporting guideline for cross-sectional studies.

### Outcomes

Trained enumerators measured children’s height using a Shorr measuring board. Stunting was defined as height-for-age *z* score less than −2 SD based on the 2006 WHO child growth reference standards.^20^

Off-track ECD was measured using the ECDI with the youngest child aged 36 to 59 months per household.^10^ Caregivers reported on whether or not their child had attained 10 developmental milestones spanning domains of literacy-numeracy, physical, socioemotional, and learning. Following prior work,^7,21^ we focused on a 5-item version of the ECDI that included the socioemotional (2 items) and learning domains (3 items). The literacy-numeracy and physical domains have been critiqued as less suitable for assessing development for children aged 36 to 59 months.^7^ For example, the physical domain item “Is your child sometimes too sick to play?” is considered more indicative of a child’s overall health than their developmental status. The other physical domain item “Can your child pick up a small object with two fingers?” is typically achieved well before 36 months of age. Following prior work,^7,21^ we created a binary indicator for off-track ECD if the child failed 2 or more items of 3 in the socioemotional domain or failed both of the 2 items in the learning domain. Using the 2 indicators for stunting and off-track ECD, we created a composite variable to classify children into 1 of 4 groups: stunted only, off-track ECD only, both stunted and off-track ECD, or neither stunted nor off-track ECD.

### Risk Factors

Based on previous studies^16,17,22,23^ and data that were available on individual-, household-, and community-level factors in the DHS and MICS, we included 17 factors from the health, early learning, safety and security, and enabling environment categories of the NCF.^1^ Health factors included basic household facilities for sanitation and drinking water as well as recent episodes of child illnesses. Early learning comprised maternal stimulation, presence of books and toys at home, and child’s attendance in early childhood education (ECE) programs. Security and safety included birth registration and child supervision. Enabling environment included factors such as maternal educational level, household wealth, and place of residence. Although nutrition and responsive caregiving are the 2 remaining components of the NCF, we were unable to include related factors due to data limitations in the DHS and MICS. More specifically, in terms of nutrition, we had initially considered dietary diversity and breastfeeding. However, most MICS surveys collect child dietary diversity, breastfeeding, and other complementary feeding indicators from children aged 2 years or younger but not children aged 3 to 4 years, which prevents analyses with the ECDI. Currently, there are no standardized or conceptually validated indicators for responsive caregiving that are collected in the DHS or MICS.^24,25^ Although we had also initially considered other potential indicators in the health domain (ie, whether childbirth was assisted by a skilled professional, antenatal care visits, and vaccinations), these variables were collected for children aged 2 years or younger only in MICS surveys. Definitions of all final risk factors included in the analysis are presented in Table 1. Most variables had low pairwise correlations, with the exception of sanitation and water source; maternal educational level and maternal age; and maternal age and marital status (eTable 2 in Supplement 1). We retained these variables with moderate to strong pairwise correlations because they represented theoretically distinct constructs with practical relevance.

**Table 1.** List of Risk Factors, Definitions, and Descriptive Statistics (N = 173 416)

Risk factor and definition	No. (%)
**NCF domain: health**	
Sanitation	
Sanitation facilities that household members use, missing %	0.1
Improved (flush toilets to piped sewer system, septic tank, or pit latrine; ventilated improved pit; pit latrine with slab; composting toilet)	76 041 (43.8)
Unimproved (flush toilets to somewhere else; pit latrines without slab or open pits; bucket or hanging toilets; open defecation)	97 247 (56.1)
Water source	
Sources of drinking water household members use, missing %	0.1
Improved (piped into dwelling, yard, plot, or neighbor; public tap or standpipe; tube well or borehole; protected well or spring; rainwater; tanker truck or cart with small tank; bottled water)	127 663 (73.6)
Unimproved (unprotected well or spring; surface water)	45 611 (26.3)
Child diarrhea	
Child had diarrhea in the last 2 wk preceding the survey, missing %	0.3
Yes	18 690 (10.8)
No	154 181 (88.9)
Child fever	
Child had fever in the last 2 wk preceding the survey, missing %	0.3
Yes	37 972 (21.9)
No	134 922 (77.8)
Child cough	
Child had cough in the last 2 wk preceding the survey, missing %	0.2
Yes	39 461 (22.8)
No	133 539 (77.0)
**NCF domain: early learning**	
Maternal stimulation	
Mother was involved in adequate or inadequate daily stimulation activities (ie, read books or looked at picture books; told stories; sang songs to or with child; took child outside the home; played with child; named, counted, or drew things for or with child) in the last 3 d preceding the survey, missing %	1.0
Adequate (involved in ≥4 stimulation activities)	37 263 (21.5)
Inadequate (involved in ≤3 stimulation activities)	134 462 (77.5)
Child books at home	
Child has child books at home, missing %	1.0
Yes (≥1 books at home)	26 298 (15.2)
No	147 087 (84.8)
Toys at home	
Child has toys at home, missing %	0.2
Yes (1-3 types of toys at home)	156 840 (90.4)
No	16 200 (9.3)
ECE attendance	
Child is currently attending ECE program, missing %	6.9
Yes	38 143 (22.0)
No	123 232 (71.1)
**NCF domain: security and safety**	
Birth registration	
Child has a birth certificate and/or their birth was registered, missing %	1.7
Yes	111 744 (64.4)
No	58 715 (33.9)
Child supervision	
Child received adequate or inadequate supervision in the last week, missing %	1.7
Adequate (child was not left alone or with another child aged <10 y)	112 524 (64.9)
Inadequate (child was left alone or with another child aged <10 y for ≥1 d)	57 875 (33.4)
**NCF domain: enabling environment**	
Maternal educational level	
Education level of mother, missing %	1.4
No or primary education	116 560 (67.2)
Secondary or higher education	54 473 (31.4)
Maternal age	
Current age of mother, missing %	0.7
15-24 y	29 894 (17.2)
25-49 y	142 368 (82.1)
Maternal marital status	
Current marital status of mother, missing %	0.3
Married or living with a partner	160 137 (92.3)
Not married (never in a union; divorced, widowed, or separated)	12 774 (7.4)
Household wealth	
Household wealth index score (a composite index of relative standard of living based on household characteristics and assets) divided into quintiles, missing %	0.0
Poorest	46 305 (26.7)
Poorer	39 039 (22.5)
Middle	33 945 (19.6)
Richer	29 454 (17.0)
Richest	24 673 (14.2)
No. of children	
No. of children aged <5 y in the household, missing %	0.0
0-2	133 170 (76.8)
≥3	40 246 (23.2)
Place of residence	
Urban or rural residency of household, missing %	0.2
Urban	50 851 (29.3)
Rural	122 181 (70.5)

Abbreviations: ECE, early childhood education; NCF, Nurturing Care Framework.

### Statistical Analysis

Statistical analysis was conducted from February to December 2024. Descriptively, the co-occurrence of stunting and off-track ECD was assessed in the pooled and country-specific samples and further stratified by country income level (low vs lower-middle income based on World Bank country income classifications), regions (East Asia and Pacific, South Asia, Middle East and North Africa, sub-Saharan Africa, and Latin America and the Caribbean), demographic and socioeconomic characteristics (ie, child’s age, household wealth, maternal educational level, and place of residence), and survey year (2013-2017 vs 2018-2020 based on median split).

Multinomial logistic regression models were used to estimate the associations between the factors and the outcomes of co-occurrence of stunting and off-track ECD, stunting only, and off-track ECD only. Initial regression models controlled for child’s age and sex, country fixed effects, survey year fixed effects, and each factor individually (eTable 3 in Supplement 1). Regression models for the main analysis controlled for child’s age and sex, country fixed effects, survey and year fixed effects and then included all factors simultaneously to estimate their independent associations with the outcome above and beyond the other factors.

For interpretation, we focused on the odds ratios (ORs) corresponding to the co-occurrence of stunting and off-track ECD with the reference category of neither stunted nor off-track ECD. In addition, we examined whether the ORs for the factors differed between co-occurrence and the other categories of stunting only and off-track ECD only. Multinomial regression analyses were repeated, alternately using stunting only and off-track ECD only as the reference categories. We denoted statistically significant differences in ORs for co-occurrence, first with stunting only and then with off-track ECD only serving as the respective reference categories. Stratified analyses were performed by country income level. We reported adjusted ORs (AORs) with 95% CIs with Bonferroni correction for multiple testing. All analyses were conducted in STATA/MP, version 16.1 (StataCorp LLC). All *P* values were from 2-sided tests and results were deemed statistically significant at *P* < .05.

## Results

**Figure 1.**
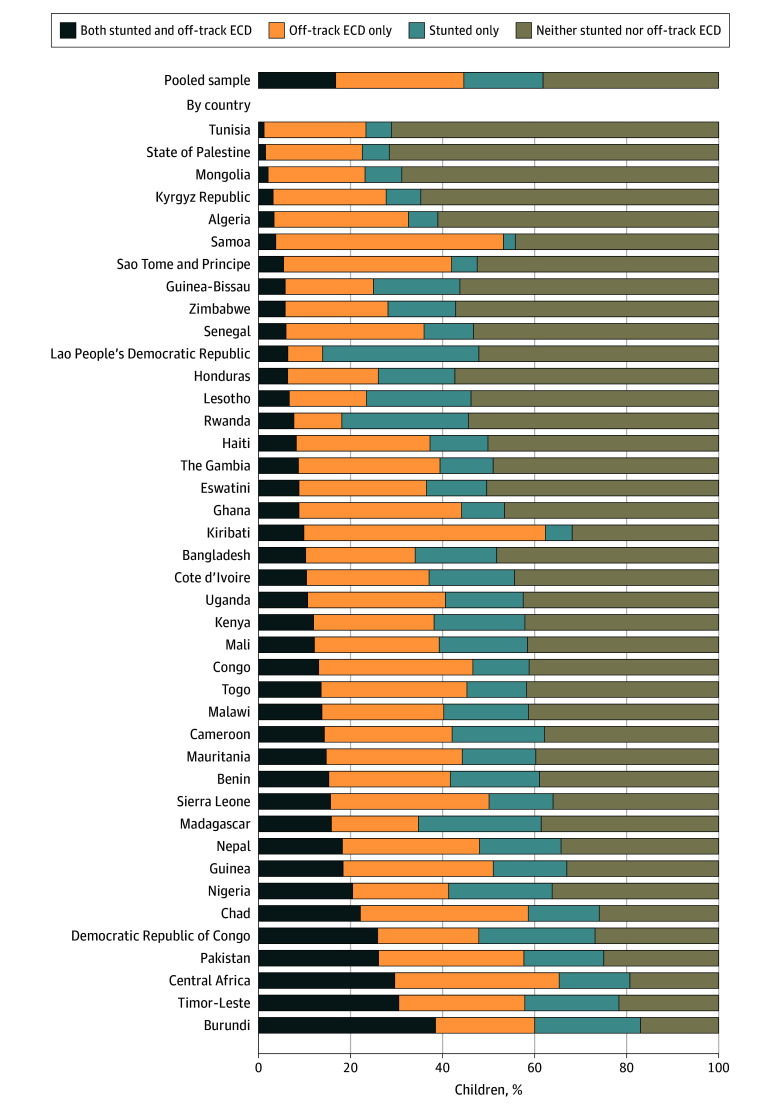
Overall Distribution of the Co-Occurrence of Stunting and Off-Track Early Childhood Development (ECD) in the Pooled and Country-Specific Samples (N = 173 416)

In the pooled sample of 173 416 children, the mean (SD) child age was 47.1 (6.8) months; 88 242 (50.9%) were boys, and 85 174 (49.1%) were girls. Overall, 59 147 children (34.1%) were reported as stunted and 77 661 (44.8%) had off-track ECD (eTable 4 in Supplement 1). Children with off-track ECD were most likely to fail in 2 particular items of the socioemotional domain: child does not kick, bite, or hit other children or adults; and child does not get distracted easily or quickly (eTable 5 in Supplement 1). When jointly considered, most young children were at risk of not thriving developmentally (ie, stunting only, off-track ECD only, or both) (Figure 1). More specifically, 17.0% (95% CI, 16.8%-17.2%) of children were both stunted and had off-track ECD (ranging from 1.4% [95% CI, 0.9%-2.2%] in Tunisia to 38.7% [95% CI, 36.7%-40.7%] in Burundi); 17.1% (95% CI, 16.9%-17.3%) were stunted only (ranging from 2.6% [95% CI, 1.7%-3.8%] in Samoa to 33.7% [95% CI, 32.3%-35.1%] in Lao People’s Democratic Republic); and 27.8% (95% CI, 27.6%-28.0%) had off-track ECD only (ranging from 7.7% [95% CI, 7.0%-8.6%] in Lao People’s Democratic Republic to 52.3% [95% CI, 48.7%-55.8%] in Kiribati). The remaining 38.1% (95% CI, 37.9%-38.4%) were neither stunted nor had off-track ECD (ranging from 17.1% [95% CI, 15.6%-18.7%] in Burundi to 71.6% [95% CI, 69.7%-73.5%] in the State of Palestine).

**Figure 2.**
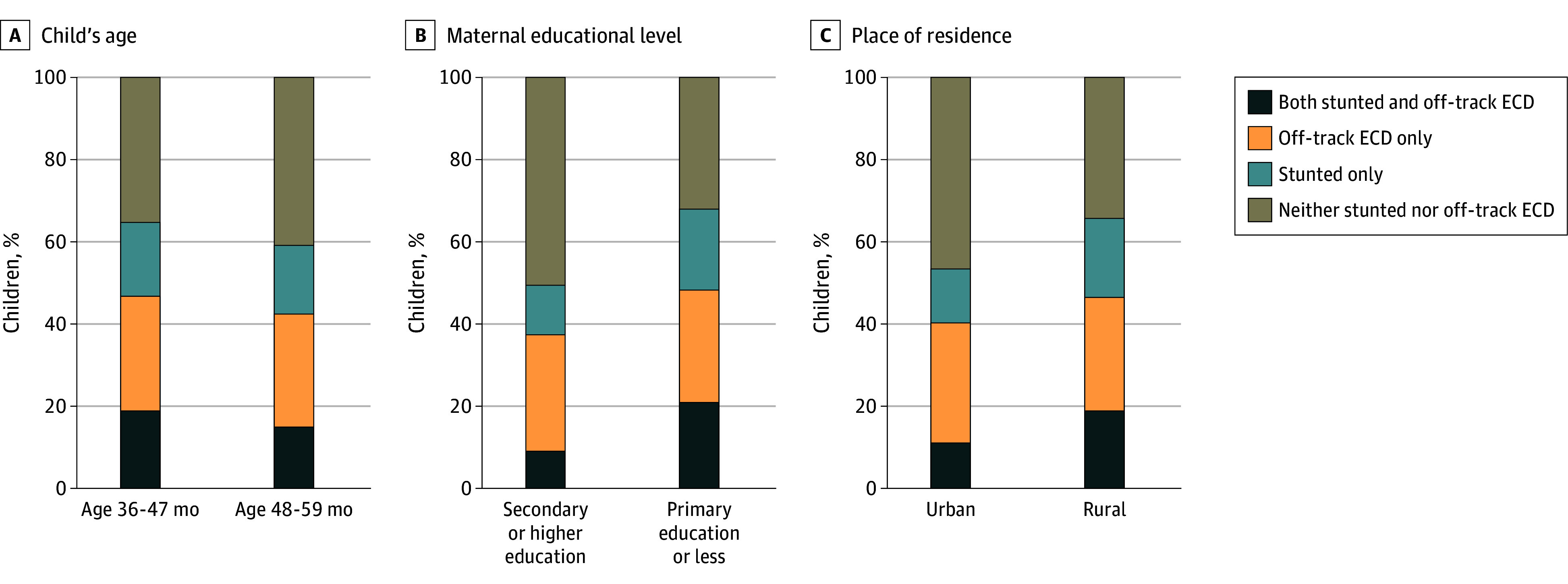
Distribution of the Co-Occurrence of Stunting and Off-Track Early Childhood Development (ECD) Stratified by Age of Children, Maternal Education, and Place of Residence (N = 173 416) The distribution of the co-occurrence of stunting and off-track ECD differed across all strata, with statistically significant χ^2^ test results for each stratification at *P* < .001.

**Figure 3.**
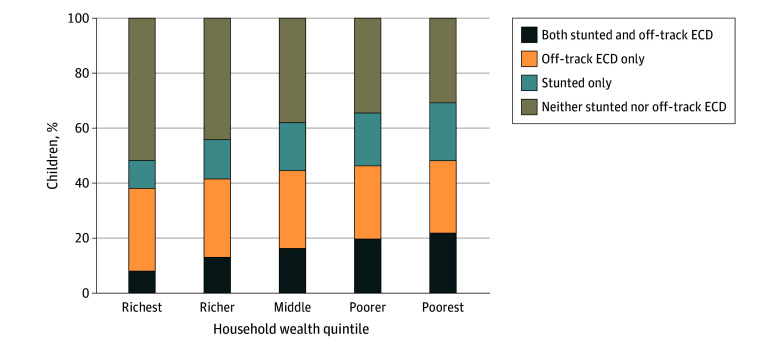
Distribution of the Co-Occurrence of Stunting and Off-Track Early Childhood Development (ECD) Stratified by Household Wealth Quintile (N = 173 416) The χ^2^ test result was statistically significant at *P* < .001.

The distribution of the co-occurrence of stunting and off-track ECD varied by country income level and across regions (eFigure 1 in Supplement 1), with higher prevalence in low-income countries (18.2% [95% CI, 17.9%-18.6%]) than lower-middle-income countries (16.5% [95% CI, 16.3%-16.7%]) and in South Asia (23.3% [95% CI, 22.9%-23.6%]) and sub-Saharan Africa (16.3% [95% CI, 16.0%-16.6%]) compared with other regions, such as the Middle East and North Africa (2.8% [95% CI, 2.5%-3.1%]) and Central Asia (3.4% [95% CI, 3.1%-3.7%]) (eTable 6 in Supplement 1). With respect to sociodemographic characteristics, a higher prevalence of co-occurrence of stunting and off-track ECD was found among younger children (19.1% [95% CI, 18.8%-19.3%] for age 36-47 months vs 14.9% [95% CI, 14.6%-15.1%] for age 48-59 months), children whose mothers had a lower educational level (20.8% [95% CI, 20.6%-21.0%] for primary education or less vs 9.1% [95% CI, 8.9%-9.4%] for secondary or higher education), and those living in rural areas (19.3% [95% CI, 19.1%-19.6%] for rural vs 11.6% [95% CI, 11.3%-11.8%] for urban) (Figure 2; eTable 7 in Supplement 1). Wealth disparities were also apparent, with a higher prevalence of the co-occurrence of stunting and off-track ECD observed among poorer households (22.1% [95% CI, 21.7%-22.5%] for the poorest wealth quintile vs 8.3% [95% CI, 8.0%-8.7%] for the richest quintile) (Figure 3). The prevalence of the co-occurrence of stunting and off-track ECD was higher for later-round surveys (17.7% [95% CI, 17.5%-17.9%] in 2018-2020 vs 15.7% [95% CI, 15.4%-16.0%] in 2013-2017) as well as for off-track ECD only (28.7% [95% CI, 28.4%-29.0%] in 2018-2020 vs 26.0% [95% CI, 25.7%-26.4%] in 2013-2017) (eFigure 2 in Supplement 1). The prevalence of stunting only was lower for later-round surveys (16.0% [95% CI, 15.8%-16.3%] in 2018-2020 vs 19.1% [95% CI, 18.8%-19.4%] in 2013-2017). The prevalence of off-track ECD, however, was similar between younger children (27.7% [95% CI, 27.4%-28.1%]) and older children (27.8% [95% CI, 27.5%-28.1%]). Stratified distributions were statistically significant based on χ^2^ tests (*P* < .001) for each sociodemographic factor.

### Risk Factors Associated With Stunting Only, Off-Track ECD Only, and Co-Occurrence

The descriptive statistics of the examined factors are presented in Table 1. In the fully adjusted model, 9 factors were significantly associated with higher odds of being stunted only compared with those who were neither stunted nor had off-track ECD (Table 2). For example, children who did not attend ECE (AOR, 1.81 [95% CI, 1.73-1.90]) or those from the poorest households (AOR, 2.18 [95% CI, 2.03-2.35]) showed a greater likelihood of being stunted only compared with the reference group. In the fully adjusted model, several factors, such as unimproved sanitation (AOR, 1.03 [95% CI, 1.00-1.07]), child cough (AOR, 1.02 [95% CI, 0.98-1.07]), and no toys at home (AOR, 0.98 [95% CI, 0.93-1.04]), were not associated with stunting only.

**Table 2.** Multinomial Logistic Regression Model Results Between Risk Factors and Co-Occurrence of Stunting and Off-Track ECD, Compared With Stunting Only and Off-Track ECD Only (N = 173 416)

Risk factor	AOR (95% CI)^a^		
	Stunted only	Off-track ECD only	Both stunted and off-track ECD
**NCF domain: health**			
Sanitation			
Improved	1 [Reference]	1 [Reference]	1 [Reference]
Unimproved	1.03 (1.00-1.07)	1.01 (0.97-1.04)	1.11 (1.07-1.16)^b^
Water source			
Improved	1 [Reference]	1 [Reference]	1 [Reference]
Unimproved	1.02 (0.98-1.06)	1.00 (0.96-1.04)	1.03 (0.99-1.08)
Child diarrhea			
No	1 [Reference]	1 [Reference]	1 [Reference]
Yes	1.26 (1.20-1.33)	1.12 (1.08-1.18)	1.38 (1.31-1.45)^b^
Child fever			
No	1 [Reference]	1 [Reference]	1 [Reference]
Yes	1.02 (0.97-1.06)	1.01 (0.97-1.04)	1.03 (0.99-1.07)
Child cough			
No	1 [Reference]	1 [Reference]	1 [Reference]
Yes	1.02 (0.98-1.07)	1.09 (1.05-1.12)	1.07 (1.02-1.11)
**NCF domain: early learning**			
Maternal stimulation			
Adequate	1 [Reference]	1 [Reference]	1 [Reference]
Inadequate	1.03 (0.99-1.08)	0.98 (0.94-1.01)	0.95 (0.91-0.99)
Child books at home			
Yes	1 [Reference]	1 [Reference]	1 [Reference]
No	1.31 (1.24-1.38)	1.19 (1.14-1.24)	1.34 (1.26-1.43)
Toys at home			
Yes	1 [Reference]	1 [Reference]	1 [Reference]
No	0.98 (0.93-1.04)	1.33 (1.26-1.40)	1.43 (1.35-1.51)^c^
ECE attendance			
Yes	1 [Reference]	1 [Reference]	1 [Reference]
No	1.81 (1.73-1.90)	1.10 (1.06-1.14)	2.22 (2.10-2.34)^b^^,^^c^
**NCF domain: safety and security**			
Birth registration			
Yes	1 [Reference]	1 [Reference]	1 [Reference]
No	1.18 (1.14-1.23)	1.07 (1.03-1.11)	1.29 (1.23-1.34)^b^^,^^c^
Child supervision			
Adequate	1 [Reference]	1 [Reference]	1 [Reference]
Inadequate	1.05 (1.01-1.09)	1.16 (1.13-1.20)	1.19 (1.14-1.23)^c^
**NCF domain: enabling environment**			
Maternal educational level			
Secondary or higher education	1 [Reference]	1 [Reference]	1 [Reference]
Primary education or less	1.36 (1.30-1.42)	1.09 (1.05-1.13)	1.44 (1.37-1.51)^b^
Maternal age			
25-49 y	1 [Reference]	1 [Reference]	1 [Reference]
15-24 y	1.18 (1.13-1.22)	0.99 (0.96-1.03)	1.18 (1.13-1.23)
Marital status			
Married or living with a partner	1 [Reference]	1 [Reference]	1 [Reference]
Not married	0.98 (0.92-1.04)	1.05 (1.00-1.10)	1.02 (0.96-1.08)
Household wealth quintile			
Richest	1 [Reference]	1 [Reference]	1 [Reference]
Richer	1.31 (1.23-1.40)	1.08 (1.02-1.13)	1.52 (1.42-1.64)^b^
Middle	1.63 (1.52-1.74)	1.19 (1.13-1.25)	1.93 (1.79-2.08)^b^^,^^c^
Poorer	1.89 (1.76-2.02)	1.20 (1.14-1.27)	2.33 (2.15-2.52)^b^^,^^c^
Poorest	2.18 (2.03-2.35)	1.33 (1.25-1.41)	2.75 (2.53-2.99)^b^^,^^c^
No. of children			
≤2	1 [Reference]	1 [Reference]	1 [Reference]
≥3	1.07 (1.03-1.11)	1.08 (1.05-1.12)	1.10 (1.05-1.14)
Residence			
Urban	1 [Reference]	1 [Reference]	1 [Reference]
Rural	1.04 (1.00-1.09)	0.99 (0.95-1.03)	0.99 (0.94-1.05)

Abbreviations: AOR, adjusted odds ratio; ECD, early childhood development; ECE, early childhood education; NCF, Nurturing Care Framework.

^a^ Model included 17 factors simultaneously to estimate the adjusted associations for each factor above and beyond the other factors. Model additionally controlled for child’s age and sex, and included country and survey year fixed effects, while adjusting for clustered SEs. “Neither stunted nor off-track ECD” served as the reference category. Two additional multinomial regressions were performed using “stunting only” and “off-track ECD only” as a reference category.

^b^ Statistically significantly higher (*P* < .05) AOR for co-occurrence group when compared with the reference group of off-track ECD only.

^c^ Statistically significantly higher (*P* < .05) AOR for co-occurrence group when compared with the reference group of stunting only.

Significant associations were found between 10 factors and having off-track ECD only. For example, no toys at home (AOR, 1.33 [95% CI, 1.26-1.40]) and belonging to the poorest households (AOR, 1.33 [95% CI, 1.25-1.41]) were associated with higher odds of having off-track ECD compared with neither (Table 2). In the fully adjusted model, several factors, such as inadequate maternal stimulation (AOR, 0.98 [95% CI, 0.94-1.01]) and younger motherhood (AOR, 0.99 [95% CI, 0.96-1.03]), were not associated with the likelihood of having off-track ECD.

These factors were most consistently associated with the co-occurrence of stunting and off-track ECD. The greatest associations were found for belonging to the poorest households (AOR, 2.75 [95% CI, 2.53-2.99]), not attending ECE (AOR, 2.22 [95% CI, 2.10-2.34]), low maternal educational level (AOR, 1.44 [95% CI, 1.37-1.51]), no toys (AOR, 1.43 [95% CI, 1.35-1.51]), and diarrhea (AOR, 1.38 [95% CI, 1.31-1.45]) (Table 2). Furthermore, the associations of many factors were significantly greater with the co-occurrence of stunting and off-track ECD compared with stunting only or off-track ECD only, such as for unimproved sanitation, child diarrhea, no toys, no ECE attendance, no birth registration, low maternal educational level, and belonging to the poorest households. Unimproved water source (AOR, 1.03 [95% CI, 0.99-1.08]), child fever (AOR, 1.03 [95% CI, 0.99-1.07]), mother being not married (AOR, 1.02 [95% CI, 0.96-1.08]), and living in rural areas (AOR, 0.99 [95% CI, 0.94-1.05]) were not associated with the co-occurrence of stunting and off-track ECD. In fact, these factors also showed null associations with the other outcome categories (ie, stunted only and off-track ECD only). On the other hand, inadequate maternal stimulation was associated with a lower odds of co-occurrence, albeit to a small magnitude (AOR, 0.95 [95% CI, 0.91-0.99]) compared with neither.

### Stratified Analysis

When stratified by country income level, the associations for all factors were consistent in their direction of association, except for maternal stimulation and place of residence (eTable 8 in Supplement 1). In low-income countries, inadequate maternal stimulation was associated with higher odds of co-occurrence compared with being neither stunted nor having off-track ECD (AOR, 1.23 [95% CI, 1.13-1.34]), whereas this association was reversed in lower-middle-income countries (AOR, 0.85 [95% CI, 0.80-0.90]). Children in rural areas showed greater likelihood of co-occurrence in low-income countries (AOR, 1.22 [95% CI, 1.11-1.35]), whereas this association was reversed in lower-middle-income countries (AOR for rural residence, 0.91 [95% CI, 0.85-0.97]).

## Discussion

In this cross-sectional study, we assessed the distribution and the risk factors associated with the co-occurrence of stunting and off-track ECD at the population level among children aged 36 to 59 months in LMICs. By examining these outcomes jointly, we sought to provide a more nuanced understanding of how children may face overlapping risks of not thriving in terms of their growth and development. Our estimates indicated that 1 in 6 children in LMICs were at risk of the double burden of stunting and off-track ECD. To our knowledge, this is the first study to quantify the co-occurrence of stunting and off-track ECD. By analyzing these indicators together, we also discovered that many children were not reaching their full potential to thrive due to a single burden as well, with a larger proportion affected by off-track ECD alone without stunting. Our results suggest that the traditional indicators of stunting and off-track ECD on their own may overlook the joint and complex developmental challenges faced by young children.

We uncovered unequal distributions in the co-occurrence of stunting and off-track ECD within and across countries. Co-occurrence was higher among poorer households, among mothers with a lower educational level, in rural settings, and in low-income countries. Co-occurrence was greatest in South Asia and sub-Saharan Africa, while relatively small in Middle and North East Africa and Central Asia. These socioeconomic gradients are consistent with regional patterns in extreme poverty, food insecurity, and limited health and social services that disproportionately constrain the poorest countries.^7,26^ These results support the validity of the indicator of co-occurrence of stunting and off-track ECD and highlight its relevance for monitoring purposes in lower-resource settings around the world.

We compared the factors of single vs double burden of stunting and off-track ECD to explore whether the risk profiles varied by the different subgroups of children at risk of not thriving. Overall, poorer household wealth, low maternal educational level, no ECE attendance, no child books at home, and child diarrhea were consistently associated with stunting only, off-track ECD only, and co-occurrence. These associations were larger for the co-occurrence group compared with the single-burden groups, suggesting that these factors pertaining to nurturing care and early-life poverty are common underlying risk factors of both stunting and off-track ECD.^27,28^ Although most of these factors have been established in prior studies on focused child growth or development separately,^17,22,23^ lack of ECE attendance emerged as a key factor across all 3 forms of suboptimal thriving, again with the strongest associations observed for co-occurrence compared with stunting only or off-track ECD only. This finding suggests the potential of ECE programs as multisectoral platforms for integrating child nutrition and ECD interventions to promote multiple child outcomes and opportunities to thrive.^29,30^ However, longitudinal evidence is needed to assess the effectiveness of ECE programs in addressing these interconnected challenges during early childhood.

Based on prior conceptual frameworks and empirical studies, we hypothesized that certain health risks (eg, poor sanitation, water source, and child illnesses) would be more distinctly associated with stunting only while early learning factors (eg, maternal stimulation and learning materials) would be more strongly associated with off-track ECD only.^6,23,27^ As expected and in line with biological mechanisms hypothesized for stunting (eg, reduced nutrient absorption and inflammation),^26^ we found that unimproved sanitation and child diarrhea were more strongly associated with co-occurrence and stunting only than off-track ECD only. However, the early learning characteristics were not consistently associated with co-occurrence and off-track ECD. Although these results upheld for lack of toys and inadequate supervision, they were not the case for inadequate maternal stimulation and lack of child books. This finding was unexpected considering the cumulative evidence supporting robust associations between these various early learning factors and ECD outcomes.^1,31,32^ Nevertheless, other studies have reported null associations between maternal stimulation and off-track ECD using the same measures and age group of children as in our study, which may be due to close engagement with other caregivers (eg, grandmothers and older siblings).^33,34^ Future research should explore in more detail the associations of maternal stimulation and the availability of books with the co-occurrence of stunting and off-track ECD.

### Limitations

This study has several limitations. First, DHS and MICS data are cross-sectional, which limited our ability to draw causal inferences. In addition, the factors in the fully adjusted model likely represent a combination of potential mediators and confounders depending on the specific factor and association under focus. Therefore, results from the adjusted models should not be interpreted as the independent association of each characteristic above and beyond other factors, but rather in a more exploratory manner. To strengthen causal inference, future studies using longitudinal data are needed to clarify temporal associations and disentangle shared risk factors associated with both stunting and off-track ECD. Second, our analysis did not include child and maternal nutrition factors (eg, diet, micronutrient status), as these were either not collected or inconsistently measured in the DHS and MICS. As a result, we were unable to determine the extent to which nutrition factors associated with co-occurrence of stunting and off-track ECD in our study, despite prior studies indicating that these factors are key determinants of early child outcomes.^23,26,27^ Similarly, we were unable to include other important factors, such as food security, caregiver’s mental health, and birth outcomes, as these were also not collected in these rounds of DHS and MICS.

## Conclusions

In this cross-sectional study using nationally representative data on young children aged 36 to 59 months in 41 LMICs, we found a substantial burden of children not thriving due to inadequate nurturing care and especially for those experiencing the double burden of stunting and off-track ECD. Our findings reinforce the NCF and its call for multisectoral interventions to holistically promote children’s potential to thrive. Longitudinal studies are needed to better understand potential causal relationships. Future research should investigate how best to design and tailor interventions to promote child thriving based on these different risk profiles.

## Notes

Supplement 1. **eTable 1.** Countries, Data Sources, Survey Years, and Sample Sizes of the Final Analytic Sample **eTable 2.** Pairwise Correlation of 17 Correlates **eTable 3.** Multinomial Logistic Regression Model Results Between Each Correlate Independently and Co-Occurrence of Stunting and Off-Track ECD, Relative to Stunting Only and Off-Track ECD Only (N=173,416) **eTable 4.** Distribution of Stunting and Off-Track Early Childhood Development in the Pooled and Country-Specific Samples (N=173,416) **eTable 5**. Proportion of Children Who Failed in Five Developmental Items Among Those With Off-Track ECD (N=77,661) **eFigure 1.** Distribution of the Co-Occurrence of Stunting and Off-Track Early Childhood Development by Country Income Grouping and by Region (N=173,416) **eFigure 2.** Distribution of the Co-Occurrence of Stunting and Off-Track Early Childhood Development Stratified by Survey Year (N=173,416) **eTable 6.** Overall Distribution of the Co-Occurrence of Stunting and Off-Track Early Childhood Development in the Pooled and Country-Specific Samples (N=173,416) **eTable 7.** Distribution of the Co-Occurrence of Stunting and Off-Track Early Childhood Development Stratified by Age of Children, Household Wealth, Maternal Education, Place of Residence, and Survey Year (N=173,416) **eTable 8.** Multinomial Logistic Regressions Between Correlates and Co-Occurrence of Stunting and Off-Track Early Childhood Development, Stratified by Income Level of Countries (N=173,416)

Supplement 2.
Data Sharing Statement
