# Supplementary material for: Co-Occurrence of Stunting and Off-Track Early Child Development in Low- and Middle-Income Countries
Source: JAMA Netw Open. 2025 Mar 4;8(3):e2462263. doi: 10.1001/jamanetworkopen.2024.62263 (PMC11880945; doi:10.1001/jamanetworkopen.2024.62263)
Supplement: Supplement 1. — eTable 1. Countries, Data Sources, Survey Years, and Sample Sizes of the Final Analytic Sample eTable 2. Pairwise Correlation of 17 Correlates eTable 3. Multinomial Logistic Regression Model Results Between Each Correlate Independently and Co-Occurrence of Stunting and Off-Track ECD, Relative to Stunting Only and Off-Track ECD Only (N=173,416) eTable 4. Distribution of Stunting and Off-Track Early Childhood Development in the Pooled and Country-Specific Samples (N=173,416) eTable 5. Proportion of Children Who Failed in Five Developmental Items Among Those With Off-Track ECD (N=77,661) eFigure 1. Distribution of the Co-Occurrence of Stunting and Off-Track Early Childhood Development by Country Income Grouping and by Region (N=173,416) eFigure 2. Distribution of the Co-Occurrence of Stunting and Off-Track Early Childhood Development Stratified by Survey Year (N=173,416) eTable 6. Overall Distribution of the Co-Occurrence of Stunting and Off-Track Early Childhood Development in the Pooled and Country-Specific Samples (N=173,416) eTable 7. Distribution of the Co-Occurrence of Stunting and Off-Track Early Childhood Development Stratified by Age of Children, Household Wealth, Maternal Education, Place of Residence, and Survey Year (N=173,416) eTable 8. Multinomial Logistic Regressions Between Correlates and Co-Occurrence of Stunting and Off-Track Early Childhood Development, Stratified by Income Level of Countries (N=173,416) [file jamanetwopen-e2462263-s001.pdf]

# Supplementary Online Content

Jeong J, Chi H, Bliznashka L, Pitchik HO, Kim R. Co-occurrence of stunting and off-track early child development in low- and middle-income countries. *JAMA Netw Open.* 2025;8(2):e2462263. doi:10.1001/jamanetworkopen.2024.62263

**eTable 1.** Countries, Data Sources, Survey Years, and Sample Sizes of the Final Analytic Sample
**eTable 2.** Pairwise Correlation of 17 Correlates
**eTable 3.** Multinomial Logistic Regression Model Results Between Each Correlate Independently and Co-Occurrence of Stunting and Off-Track ECD, Relative to Stunting Only and Off-Track ECD Only (N=173,416)
**eTable 4.** Distribution of Stunting and Off-Track Early Childhood Development in the Pooled and Country-Specific Samples (N=173,416)
**eTable 5**. Proportion of Children Who Failed in Five Developmental Items Among Those With Off-Track ECD (N=77,661)
**eFigure 1.** Distribution of the Co-Occurrence of Stunting and Off-Track Early Childhood Development by Country Income Grouping and by Region (N=173,416)
**eFigure 2.** Distribution of the Co-Occurrence of Stunting and Off-Track Early Childhood Development Stratified by Survey Year (N=173,416)
**eTable 6.** Overall Distribution of the Co-Occurrence of Stunting and Off-Track Early Childhood Development in the Pooled and Country-Specific Samples (N=173,416)
**eTable 7.** Distribution of the Co-Occurrence of Stunting and Off-Track Early Childhood Development Stratified by Age of Children, Household Wealth, Maternal Education, Place of Residence, and Survey Year (N=173,416)
**eTable 8.** Multinomial Logistic Regressions Between Correlates and Co-Occurrence of Stunting and Off-Track Early Childhood Development, Stratified by Income Level of Countries (N=173,416)

This supplementary material has been provided by the authors to give readers additional information about their work.

© 2025 Jeong J et al. *JAMA Network Open.*

**eTable 1.** Countries, Data Sources, Survey Years, and Sample Sizes of the Final Analytic Sample

| Region | Income level | Survey year | Country | Data source (phase/round) | Sample size |
|---|---|---|---|---|---|
| Central Asia | Lower-middle | 2018 | Kyrgyz Republic | MICS6 | 1,279 |
| East Asia and Pacific | Lower-middle | 2016 | Timore Leste | DHS7 | 805 |
| | | 2017 | Lao PDR | MICS6 | 4,301 |
| | | 2018 | Mongolia | MICS6 | 2,309 |
| | | 2018-2019 | Kiribati | MICS6 | 767 |
| | | 2019-2020 | Samoa | MICS6 | 902 |
| Latin America and the Caribbean | Lower-middle | 2016-2017 | Haiti | DHS7 | 1,418 |
| | | 2019 | Honduras | MICS6 | 3,247 |
| Middle East and North Africa | Lower-middle | 2018 | Tunisia | MICS6 | 1,395 |
| | | 2018-2019 | Algeria | MICS6 | 5,597 |
| | | 2019-2020 | State of Palestine | MICS6 | 2,151 |
| South Asia | Lower-middle | 2016-2020 | Pakistan | MICS6 | 43,522 |
| | | 2019 | Bangladesh | MICS6 | 8,873 |
| | | 2019 | Nepal | MICS6 | 2,692 |
| Sub-Saharan Africa | Low | 2015 | Mali | MICS5 | 5,357 |
| | | 2016 | Uganda | DHS7 | 1,596 |
| | | 2016-2017 | Burundi | DHS7 | 2,315 |
| | | 2017 | Sierra Leone | MICS6 | 3851 |
| | | 2017 | Togo | MICS6 | 1,766 |
| | | 2017-2018 | Congo DR | MICS6 | 7,649 |
| | | 2018 | Madagascar | MICS6 | 4,425 |
| | | 2018 | The Gambia | MICS6 | 3,780 |
| | | 2018-2019 | Central African Republic | MICS6 | 3,090 |
| | | 2018-2019 | Guinea-Bissau | MICS6 | 2716 |
| | | 2019 | Chad | MICS6 | 8,688 |
| | | 2019-2020 | Malawi | MICS6 | 5,247 |
| | | 2019-2020 | Rwanda | DHS7 | 1,447 |
| | Lower-middle | 2011-2014 | Kenya | MICS5 | 795 |

© 2025 Jeong J et al. *JAMA Network Open.*

| | | | | |
|---|---|---|---|---|
| | 2014 | Cameroon | MICS5 | 2,380 |
| | 2014 | Eswatini | MICS5 | 780 |
| | 2014-2015 | Congo | MICS5 | 3,148 |
| | 2015 | Mauritania | MICS5 | 3,491 |
| | 2016 | Cote d'Ivoire | MICS5 | 3,080 |
| | 2016 | Guinea | MICS5 | 2,722 |
| | 2016-2017 | Nigeria | MICS5 | 10,225 |
| | 2017-2018 | Benin | DHS7 | 3,794 |
| | 2017-2018 | Ghana | MICS6 | 6,154 |
| | 2018 | Lesotho | MICS6 | 905 |
| | 2019 | Sao Tome and Principe | MICS6 | 662 |
| | 2019 | Senegal | DHS7 | 1,985 |
| | 2019 | Zimbabwe | MICS6 | 2,110 |

Note. MICS = Multiple Indicator Cluster Surveys; DHS = Demographic and Health Surveys; Lao PDR = Lao People's Democratic Republic; Congo DR = Democratic Republic of Congo. For Kenya, data from Nyanza province (MICS 2011) was excluded because a different set of 18 ECDI items were collected.

© 2025 Jeong J et al. *JAMA Network Open.*

**eTable 2.** Pairwise Correlation of 17 Correlates

| | Sanitation | Water source | Child diarrhea | Child fever | Child cough | Maternal stimulation | Child books at home | Toys at home | ECE attendance | Birth registration | Child supervision | Maternal education | Maternal age | Maternal marital status | Household wealth | Number of children | Place of residence |
|---|---|---|---|---|---|---|---|---|---|---|---|---|---|---|---|---|---|
| Sanitation | 1.00 | | | | | | | | | | | | | | | | |
| Water source | 0.81*** | 1.00 | | | | | | | | | | | | | | | |
| Child diarrhea | 0.00 | 0.00 | 1.00 | | | | | | | | | | | | | | |
| Child fever | 0.00 | 0.00 | 0.22*** | 1.00 | | | | | | | | | | | | | |
| Child cough | 0.00 | 0.00 | 0.19*** | 0.30*** | 1.00 | | | | | | | | | | | | |
| Maternal stimulation | 0.00 | 0.00 | 0.01*** | 0.02*** | 0.02*** | 1.00 | | | | | | | | | | | |
| Child books at home | 0.01*** | 0.00 | 0.02*** | 0.02*** | 0.02*** | 0.02*** | 1.00 | | | | | | | | | | |
| Toys at home | 0.01*** | 0.00 | 0.05*** | 0.06*** | 0.05*** | 0.02*** | 0.05*** | 1.00 | | | | | | | | | |
| ECE attendance | 0.02*** | 0.01** | 0.00 | 0.00 | -.01* | 0.02*** | 0.02*** | 0.00 | 1.00 | | | | | | | | |
| Birth registration | 0.02*** | 0.02*** | 0.04*** | 0.04*** | 0.04*** | 0.02*** | 0.01*** | 0.03*** | 0.01*** | 1.00 | | | | | | | |
| Child supervision | 0.00 | 0.00 | 0.05*** | 0.07*** | 0.04*** | 0.02*** | 0.03*** | 0.04*** | 0.02*** | 0.04*** | 1.00 | | | | | | |
| Maternal education | 0.01** | 0.00 | 0.00 | 0.00 | 0.00 | 0.00 | 0.00 | 0.00 | -0.02*** | 0.00 | 0.00 | 1.00 | | | | | |
| Maternal age | 0.00 | 0.00 | 0.00 | 0.00 | 0.00 | 0.01*** | 0.00 | 0.00 | -0.01** | 0.00 | 0.00 | 0.69*** | 1.00 | | | | |
| Maternal marital status | 0.00 | 0.00 | 0.00 | 0.00 | 0.00 | 0.00 | 0.00 | 0.01** | -0.01* | 0.00 | 0.00 | 0.41*** | 0.59*** | 1.00 | | | |
| Household wealth | 0.06*** | 0.03*** | 0.01* | 0.00 | 0.00 | 0.01** | 0.07*** | 0.02*** | 0.06*** | 0.04*** | 0.02*** | 0.03*** | 0.00 | 0.00 | 1.00 | | |
| Number of children | 0.00 | 0.00 | 0.01** | 0.01** | 0.01** | 0.02*** | 0.03*** | 0.01*** | -0.02*** | 0.01*** | 0.02*** | 0.00 | 0.00 | -0.01** | 0.03*** | 1.00 | |
| Place of residence | -0.01*** | 0.00 | 0.00 | 0.00 | 0.00 | 0.00 | 0.00 | 0.00 | -0.01*** | 0.00 | -0.01* | 0.01** | 0.00 | 0.00 | 0.05*** | 0.00 | 1.00 |

Note. P-value is signified with * at p<0.05; ** at p<0.01; *** at p<0.001.

© 2025 Jeong J et al. *JAMA Network Open.*

**eTable 3.** Multinomial Logistic Regression Model Results Between Each Correlate Independently and Co-Occurrence of Stunting and Off-Track ECD, Relative to Stunting Only and Off-Track ECD Only (N=173,416)

| NCF domain | Correlate | AOR (95% CI) | | |
|---|---|---|---|---|
| | | Stunted only | Off-track ECD only | Both stunted and off-track ECD |
| Health | Sanitation | | | |
| | Improved | Reference | Reference | Reference |
| | Unimproved | 1.52 (1.47, 1.58) *** | 1.17 (1.13, 1.21) *** | 1.78 (1.71, 1.85) *** [a,b] |
| | Water source | | | |
| | Improved | Reference | Reference | Reference |
| | Unimproved | 1.35 (1.30, 1.40) *** | 1.12 (1.08, 1.17) *** | 1.41 (1.35, 1.48) *** [a,b] |
| | Child diarrhea | | | |
| | No | Reference | Reference | Reference |
| | Yes | 1.36 (1.29, 1.43) *** | 1.18 (1.12, 1.23) *** | 1.51 (1.44, 1.59) *** [a,b] |
| | Child fever | | | |
| | No | Reference | Reference | Reference |
| | Yes | 1.09 (1.05, 1.13) *** | 1.07 (1.03, 1.10) ** | 1.15 (1.11, 1.19) *** [a,b] |
| | Child cough | | | |
| | No | Reference | Reference | Reference |
| | Yes | 1.02 (0.98, 1.05) | 1.08 (1.05, 1.12) *** | 1.08 (1.04, 1.12) ** [a] |
| Early learning | Maternal stimulation | | | |
| | Adequate | Reference | Reference | Reference |
| | Inadequate | 1.41 (1.35, 1.47) *** | 1.10 (1.07, 1.14) *** | 1.35 (1.29, 1.42) *** [b] |
| | Child books at home | | | |
| | Yes | Reference | Reference | Reference |
| | No | 2.19 (2.09, 2.31) *** | 1.38 (1.33, 1.44) *** | 2.50 (2.35, 2.66) *** [a,b] |
| | Toys at home | | | |
| | Yes | Reference | Reference | Reference |
| | No | 1.16 (1.10, 1.23) *** | 1.41 (1.34, 1.48) *** | 1.70 (1.61, 1.80) *** [a,b] |
| | ECE attendance | | | |
| | Yes | Reference | Reference | Reference |
| | No | 2.61 (2.50, 2.72) *** | 1.28 (1.23, 1.32) *** | 3.49 (3.31, 3.68) *** [a,b] |
| Safety and security | Birth registration | | | |
| | Yes | Reference | Reference | Reference |
| | No | 1.61 (1.55, 1.67) *** | 1.19 (1.15, 1.24) *** | 1.85 (1.77, 1.93) *** [a,b] |
| | Child supervision | | | |
| | Adequate | Reference | Reference | Reference |
| | Inadequate | 1.15 (1.11, 1.19) *** | 1.19 (1.16, 1.23) *** | 1.30 (1.25, 1.35) *** [a,b] |
| Enabling environment | Maternal education | | | |
| | Secondary to higher | Reference | Reference | Reference |
| | No or primary | 2.20 (2.12, 2.29) *** | 1.31 (1.27, 1.35) *** | 2.66 (2.55, 2.79) *** [a,b] |
| | Maternal age | | | |
| | 25-49 years | Reference | Reference | Reference |

© 2025 Jeong J et al. *JAMA Network Open.*

| | | | | |
|---|---|---|---|---|
| | 15-24 years | 1.21 (1.17, 1.25) *** | 1.01 (0.97, 1.04) | 1.22 (1.17, 1.27) *** [b] |
| | Marital status | | | |
| | Married/living with a partner | Reference | Reference | Reference |
| | Not married | 0.95 (0.89, 1.00) | 1.03 (0.98, 1.08) | 0.98 (0.92, 1.05) |
| | Household wealth quintile | | | |
| | Richest | Reference | Reference | Reference |
| | Richer | 1.63 (1.53, 1.74) *** | 1.16 (1.11, 1.21) *** | 1.96 (1.82, 2.10) *** [a, b] |
| | Middle | 2.32 (2.18, 2.46) *** | 1.34 (1.28, 1.40) *** | 2.91 (2.72, 3.11) *** [a, b] |
| | Poorer | 2.95 (2.77, 3.13) *** | 1.40 (1.34, 1.47) *** | 3.94 (3.68, 4.22) *** [a, b] |
| | Poorest | 3.71 (3.50, 3.95) *** | 1.60 (1.53, 1.68) *** | 5.26 (4.91, 5.63) *** [a, b] |
| | Number of children | | | |
| | 0-2 | Reference | Reference | Reference |
| | 3+ | 1.17 (1.13, 1.22) *** | 1.12 (1.09, 1.16) *** | 1.19 (1.14, 1.24) *** |
| | Residence | | | |
| | Urban | Reference | Reference | Reference |
| | Rural | 1.77 (1.70, 1.85) *** | 1.21 (1.17, 1.25) *** | 1.93 (1.83, 2.02) *** [a, b] |

Note. These results are from models that estimated the associations of each correlate independently (not simultaneously with all correlates included in the same model). Models also adjusted for child's age, sex, and including country and survey year fixed effects, while adjusting for clustered standard errors. "Neither stunted nor off-track ECD" served as the reference category. P-value adjusted for Bonferroni correction and is denoted with * at p<0.05; ** at p<0.01; *** at p<0.001. AOR = adjusted odds ratio. CI = confidence interval. ECD = early childhood development. ECE = early childhood education. Two additional multinomial regressions were performed using "stunting only" and "off-track ECD only" as a reference category. Statistically significant differences (p<0.05) of the coefficients for the co-occurrence group using the reference groups of stunting only and off-track ECD only are indicated with a and b, respectively.

© 2025 Jeong J et al. *JAMA Network Open.*

**eTable 4.** Distribution of Stunting and Off-Track Early Childhood Development in the Pooled and Country-Specific Samples (N=173,416)

| Region | Income level | Survey year | Country | N (%) | |
|---|---|---|---|---|---|
| | | | | **Stunting** | **Off-track ECD** |
| Pooled sample | | | | 59,147 (34.1) | 77,661 (44.8) |
| Central Asia | Lower-middle | 2018 | Kyrgyz Republic | 139 (10.9) | 356 (27.8) |
| East Asia and Pacific | Lower-middle | 2016 | Timore Leste | 412 (51.2) | 466 (57.9) |
| | | 2017 | Lao PDR | 1,727 (40.2) | 610 (14.2) |
| | | 2018 | Mongolia | 237 (10.3) | 536 (23.2) |
| | | 2018-2019 | Kiribati | 121 (15.8) | 479 (62.5) |
| | | 2019-2020 | Samoa | 59 (6.5) | 481 (53.3) |
| Latin America and the Caribbean | Lower-middle | 2016-2017 | Haiti | 297 (20.9) | 530 (37.4) |
| | | 2019 | Honduras | 750 (23.1) | 853 (26.3) |
| Middle East and North Africa | Lower-middle | 2018 | Tunisia | 98 (7.0) | 326 (23.4) |
| | | 2018-2019 | Algeria | 548 (9.8) | 1,833 (32.8) |
| | | 2019-2020 | State of Palestine | 162 (7.5) | 487 (22.6) |
| South Asia | Lower-middle | 2016-2020 | Pakistan | 18,927 (43.5) | 25,116 (57.7) |
| | | 2019 | Bangladesh | 2,490 (28.1) | 3,031 (34.2) |
| | | 2019 | Nepal | 970 (36.0) | 1,294 (48.1) |
| Sub-Saharan Africa | Low | 2015 | Mali | 1,689 (31.5) | 2,112 (39.4) |
| | | 2016 | Uganda | 441 (27.6) | 650 (40.7) |
| | | 2016-2017 | Burundi | 1,429 (61.7) | 1,387 (59.9) |
| | | 2017 | Sierra Leone | 1,143 (29.7) | 1,932 (50.2) |
| | | 2017 | Togo | 474 (26.8) | 801 (45.4) |
| | | 2017-2018 | Congo DR | 3,919 (51.2) | 3,658 (47.8) |
| | | 2018 | Madagascar | 1,884 (42.6) | 1,546 (34.9) |
| | | 2018 | The Gambia | 767 (20.3) | 1,496 (39.6) |
| | | 2018-2019 | Central African Republic | 1,398 (45.2) | 2,019 (65.3) |
| | | 2018-2019 | Guinea-Bissau | 668 (24.6) | 684 (25.2) |
| | | 2019 | Chad | 3,272 (37.7) | 5,100 (58.7) |
| | | 2019-2020 | Malawi | 1,691 (32.2) | 2,119 (40.4) |
| | | 2019-2020 | Rwanda | 509 (35.2) | 265 (18.3) |
| | Lower-middle | 2011-2014 | Kenya | 253 (31.8) | 304 (38.2) |
| | | 2014 | Cameroon | 823 (34.6) | 1,002 (42.1) |
| | | 2014 | Eswatini | 171 (21.9) | 286 (36.7) |
| | | 2014-2015 | Congo | 802 (25.5) | 1,467 (46.6) |
| | | 2015 | Mauritania | 1,070 (30.7) | 1,551 (44.4) |
| | | 2016 | Cote d'Ivoire | 899 (29.2) | 1,144 (37.1) |
| | | 2016 | Guinea | 946 (34.8) | 1,389 (51.0) |
| | | 2016-2017 | Nigeria | 4,405 (43.1) | 4,239 (41.5) |
| | | 2017-2018 | Benin | 1,313 (34.6) | 1,589 (41.9) |

© 2025 Jeong J et al. *JAMA Network Open.*

| | | | | |
|---|---|---|---|---|
| | 2017-2018 | Ghana | 1,132 (18.4) | 2,718 (44.2) |
| | 2018 | Lesotho | 267 (29.5) | 213 (23.5) |
| | 2019 | Sao Tome and Principe | 74 (11.2) | 278 (42.0) |
| | 2019 | Senegal | 334 (16.8) | 717 (36.1) |
| | 2019 | Zimbabwe | 437 (20.7) | 597 (28.3) |
| *By income level* | | | | |
| Low income | | | 19,284 (37.1) | 23,769 (45.8) |
| Lower-middle income | | | 39,863 (32.8) | 53,892 (44.4) |
| *By region* | | | | |
| Central Asia | | | 139 (10.9) | 356 (27.8) |
| East Asia and Pacific | | | 2,556 (28.1) | 2,572 (28.3) |
| Latin America and the Caribbean | | | 1,047 (22.4) | 1,383 (29.7) |
| Middle East and North Africa | | | 808 (8.8) | 2,646 (28.9) |
| South Asia | | | 22,387 (40.6) | 29,441 (53.4) |
| Sub-Saharan Africa | | | 32,210 (34.2) | 41,263 (43.8) |

Note. ECD = early childhood development. Lao PDR = Lao People’s Democratic Republic; Congo DR = Democratic Republic of Congo.

© 2025 Jeong J et al. *JAMA Network Open.*

**eTable 5**. Proportion of Children Who Failed in Five Developmental Items Among Those With Off-Track ECD (N=77,661)

| **Developmental domains** | **Children with off-track ECD (N=77,661)** | |
|---|---|---|
| ***Socioemotional domain*** | **Failed, N (%)** | **Passed, N (%)** |
| Child gets along well with other children | 17,851 (23.0) | 59,810 (77.0) |
| Child does not kick, bite, or hit other children or adults | 57,412 (73.9) | 20,249 (26.1) |
| Child does not get distracted easily or quickly | 58,074 (74.8) | 19,587 (25.2) |
| ***Learning domain*** | **Failed, N (%)** | **Passed, N (%)** |
| Child follows simple directions on how to do something correctly | 34,429 (44.3) | 43,232 (55.7) |
| Child can work on a task, including play tasks independently | 37,816 (48.7) | 39,845 (51.3) |

Note. For the socioemotional domain item "Child does not kick, bite, or hit other children or adults", those who failed in this item mean that they were reported to kick, bite, or hit others. Similarly, those who failed in the item "Child does not get distracted easily or quickly" mean that they were reported to be distracted easily or quickly.

© 2025 Jeong J et al. *JAMA Network Open.*

**eFigure 1.** Distribution of the Co-Occurrence of Stunting and Off-Track Early Childhood Development by Country Income Grouping and by Region (N=173,416)

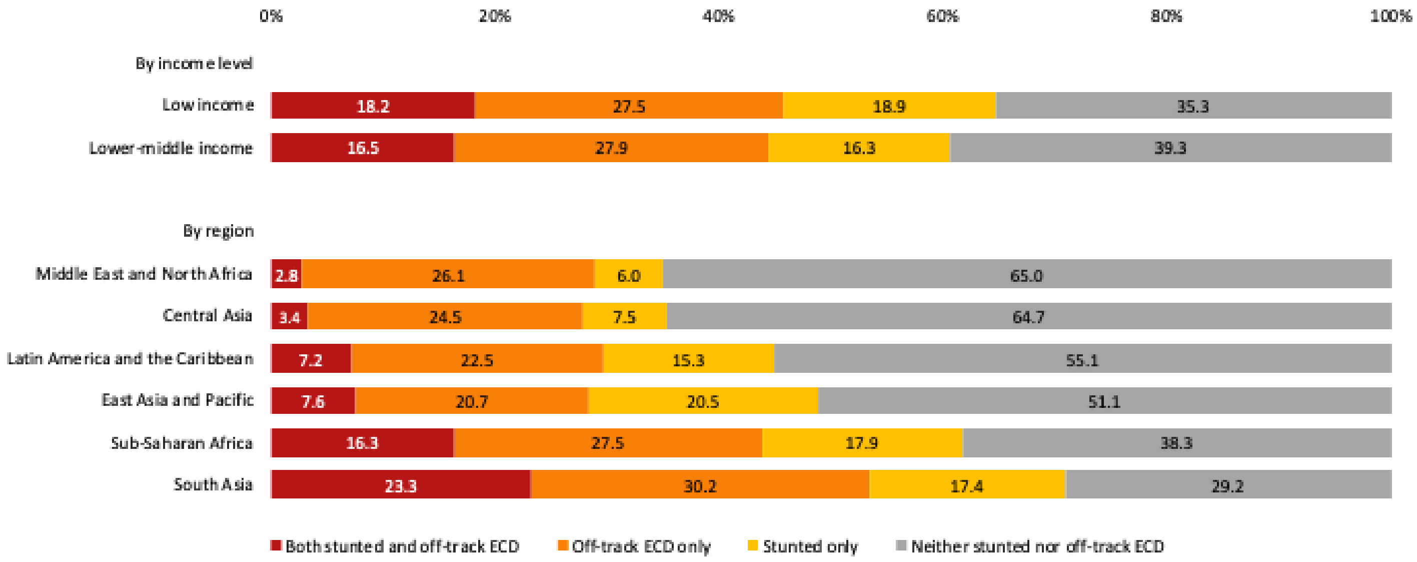


© 2025 Jeong J et al. *JAMA Network Open.*

**eFigure 2.** Distribution of the Co-Occurrence of Stunting and Off-Track Early Childhood Development Stratified by Survey Year (N=173,416)

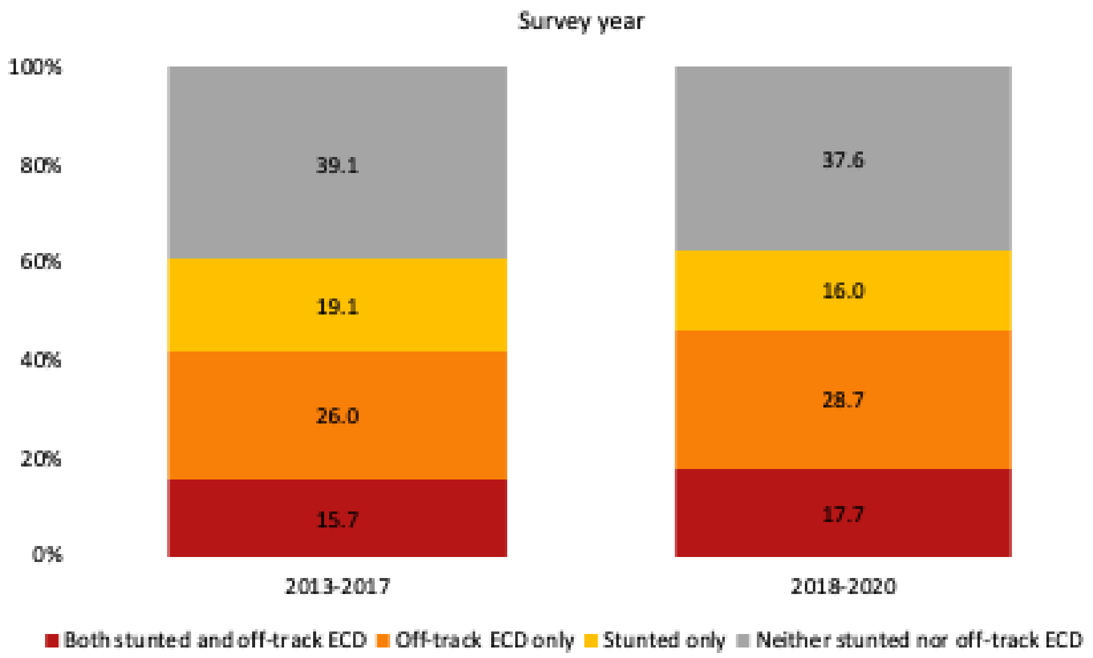


Note. Chi-square test result was statistically significant at p-value<0.001.

© 2025 Jeong J et al. *JAMA Network Open.*

**eTable 6.** Overall Distribution of the Co-Occurrence of Stunting and Off-Track Early Childhood Development in the Pooled and Country-Specific Samples (N=173,416)

| | **N (%)** | | | |
|---|---|---|---|---|
| | **Both stunted and off-track ECD** | **Off-track ECD only** | **Stunted only** | **Neither stunted nor off-track ECD** |
| Pooled sample | 29,502 (17.0) | 48,159 (27.8) | 29,645 (17.1) | 66,110 (38.1) |
| *By country* | | | | |
| Algeria | 198 (3.5) | 1,635 (29.2) | 350 (6.3) | 3,414 (61.0) |
| Bangladesh | 926 (10.4) | 2,105 (23.7) | 1,564 (17.6) | 4,278 (48.2) |
| Benin | 586 (15.4) | 1,003 (26.4) | 727 (19.2) | 1,478 (39.0) |
| Burundi | 896 (38.7) | 491 (21.2) | 533 (23.0) | 395 (17.1) |
| Cameroon | 346 (14.5) | 656 (27.6) | 477 (20.0) | 901 (37.9) |
| Central Africa | 920 (29.8) | 1,099 (35.6) | 478 (15.5) | 593 (19.2) |
| Chad | 1,936 (22.3) | 3,164 (36.4) | 1,336 (15.4) | 2,252 (25.9) |
| Congo | 414 (13.2) | 1,053 (33.4) | 388 (12.3) | 1,293 (41.1) |
| Cote d'Ivoire | 330 (10.7) | 814 (26.4) | 569 (18.5) | 1,367 (44.4) |
| Congo DR | 1,986 (26.0) | 1,672 (21.9) | 1,933 (25.3) | 2,058 (26.9) |
| Eswatini | 70 (9.0) | 216 (27.7) | 101 (12.9) | 393 (50.4) |
| Ghana | 556 (9.0) | 2,162 (35.1) | 576 (9.4) | 2,860 (46.5) |
| Guinea | 509 (18.7) | 880 (32.3) | 437 (16.1) | 896 (32.9) |
| Guinea-Bissau | 164 (6.0) | 520 (19.1) | 504 (18.6) | 1,528 (56.3) |
| Haiti | 119 (8.4) | 411 (29.0) | 178 (12.6) | 710 (50.1) |
| Honduras | 215 (6.6) | 638 (19.6) | 535 (16.5) | 1,859 (57.3) |
| Kenya | 96 (12.1) | 208 (26.2) | 157 (19.7) | 334 (42.0) |
| Kiribati | 78 (10.2) | 401 (52.3) | 43 (5.6) | 245 (31.9) |
| Kyrgyz Republic | 43 (3.4) | 313 (24.5) | 96 (7.5) | 827 (64.7) |
| Lao PDR | 278 (6.5) | 332 (7.7) | 1,449 (33.7) | 2,242 (52.1) |
| Lesotho | 62 (6.9) | 151 (16.7) | 205 (22.7) | 487 (53.8) |
| Madagascar | 707 (16.0) | 839 (19.0) | 1,177 (26.6) | 1,702 (38.5) |
| Malawi | 736 (14.0) | 1,383 (26.4) | 955 (18.2) | 2,173 (41.4) |
| Mali | 663 (12.4) | 1,449 (27.0) | 1,026 (19.2) | 2,219 (41.4) |
| Mauritania | 517 (14.8) | 1,034 (29.6) | 553 (15.8) | 1,387 (39.7) |
| Mongolia | 51 (2.2) | 485 (21.0) | 186 (8.1) | 1,587 (68.7) |
| Nepal | 495 (18.4) | 799 (29.7) | 475 (17.6) | 923 (34.3) |
| Nigeria | 2,119 (20.7) | 2,120 (20.7) | 2,286 (22.4) | 3,700 (36.2) |
| Pakistan | 11,388 (26.2) | 13,728 (31.5) | 7,539 (17.3) | 10,867 (25.0) |
| Rwanda | 113 (7.8) | 152 (10.5) | 396 (27.4) | 786 (54.3) |

© 2025 Jeong J et al. *JAMA Network Open.*

| | | | | |
|---|---|---|---|---|
| Samoa | 36 (4.0) | 445 (49.3) | 23 (2.5) | 398 (44.1) |
| Sao Tome and Principe | 37 (5.6) | 241 (36.4) | 37 (5.6) | 347 (52.4) |
| Senegal | 123 (6.2) | 594 (29.9) | 211 (10.6) | 1,057 (53.2) |
| Sierra Leone | 608 (15.8) | 1,324 (34.4) | 535 (13.9) | 1,384 (35.9) |
| State of Palestine | 39 (1.8) | 448 (20.8) | 123 (5.7) | 1,541 (71.6) |
| The Gambia | 330 (8.7) | 1,166 (30.8) | 437 (11.6) | 1,847 (48.9) |
| Timore Leste | 247 (30.7) | 219 (27.2) | 165 (20.5) | 174 (21.6) |
| Togo | 244 (13.8) | 557 (31.5) | 230 (13.0) | 735 (41.6) |
| Tunisia | 20 (1.4) | 306 (21.9) | 78 (5.6) | 991 (71.0) |
| Uganda | 173 (10.8) | 477 (29.9) | 268 (16.8) | 678 (42.5) |
| Zimbabwe | 128 (6.1) | 469 (22.2) | 309 (14.6) | 1,204 (57.1) |
| *By income level* | | | | |
| Low income | 9,476 (18.2) | 14,293 (27.5) | 9,808 (18.9) | 18,350 (35.3) |
| Lower-middle income | 20,026 (16.5) | 33,866 (27.9) | 19,837 (16.3) | 47,760 (39.3) |
| *By region* | | | | |
| Central Asia | 43 (3.4) | 313 (24.5) | 96 (7.5) | 827 (64.7) |
| East Asia and Pacific | 690 (7.6) | 1,882 (20.7) | 1,866 (20.5) | 4,646 (51.1) |
| Latin America and the Caribbean | 334 (7.2) | 1,049 (22.5) | 713 (15.3) | 2,569 (55.1) |
| Middle East and North Africa | 257 (2.8) | 2,389 (26.1) | 551 (6.0) | 5,946 (65.0) |
| South Asia | 12,809 (23.3) | 16,632 (30.2) | 9,578 (17.4) | 16,068 (29.2) |
| Sub-Saharan Africa | 15,369 (16.3) | 25,894 (27.5) | 16,841 (17.9) | 36,054 (38.3) |

© 2025 Jeong J et al. *JAMA Network Open.*

**eTable 7.** Distribution of the Co-Occurrence of Stunting and Off-Track Early Childhood Development Stratified by Age of Children, Household Wealth, Maternal Education, Place of Residence, and Survey Year (N=173,416)

| | N (%) | | | | P-value |
|---|---|---|---|---|---|
| | **Both stunted and off-track ECD** | **Off-track ECD only** | **Stunted only** | **Neither stunted nor off-track ECD** | |
| *By age* | | | | | <0.001 |
| 36-47 months | 16,901 (19.1) | 24,602 (27.7) | 15,821 (17.8) | 31,333 (35.3) | |
| 48-59 months | 12,601 (14.9) | 23,557 (27.8) | 13,824 (16.3) | 34,777 (41.0) | |
| *By wealth* | | | | | <0.001 |
| Richest | 2,055 (8.3) | 7,357 (29.8) | 2,572 (10.4) | 12,689 (51.4) | |
| Richer | 3,890 (13.2) | 8,470 (28.8) | 4,148 (14.1) | 12,946 (44.0) | |
| Middle | 5,588 (16.5) | 9,681 (28.5) | 5,757 (17.0) | 12,919 (38.1) | |
| Poorer | 7,732 (19.8) | 10,389 (26.6) | 7,519 (19.3) | 13,399 (34.3) | |
| Poorest | 10,237 (22.1) | 12,262 (26.5) | 9,649 (20.8) | 14,157 (30.6) | |
| *By education* | | | | | <0.001 |
| Secondary to higher | 4,975 (9.1) | 15,390 (28.3) | 6,509 (11.9) | 27,599 (50.7) | |
| No education to primary | 24,225 (20.8) | 32,017 (27.5) | 22,810 (19.6) | 37,508 (32.2) | |
| *By residence* | | | | | <0.001 |
| Urban | 5,880 (11.6) | 14,737 (29.0) | 6,439 (12.7) | 23,795 (46.8) | |
| Rural | 23,614 (19.3) | 33,341 (27.3) | 23,190 (19.0) | 42,036 (34.4) | |
| *By survey year* | | | | | <0.001 |
| 2013-2017 | 9,396 (15.7) | 15,546 (26.0) | 11,407 (19.1) | 23,371 (39.1) | |
| 2018-2020 | 20,106 (17.7) | 32,613 (28.7) | 18,238 (16.0) | 42,739 (37.6) | |

Note. Chi-square test results showed statistical significance for each stratification, presented with p-values.

© 2025 Jeong J et al. *JAMA Network Open.*

**eTable 8.** Multinomial Logistic Regressions Between Correlates and Co-Occurrence of Stunting and Off-Track Early Childhood Development, Stratified by Income Level of Countries (N=173,416)

| NCF domain | Correlate | Low-income countries (13 countries, N=51,927) AOR (95% CI) | | | Lower-middle income countries (28 countries, N=121,489) AOR (95% CI) | | |
|---|---|---|---|---|---|---|---|
| | | **Stunted only** | **Off-track ECD only** | **Both stunted and off-track ECD** | **Stunted only** | **Off-track ECD only** | **Both stunted and off-track ECD** |
| Health | Sanitation | | | | | | |
| | Improved | Reference | Reference | Reference | Reference | Reference | Reference |
| | Unimproved | 1.11 (1.03, 1.20) | 0.99 (0.92, 1.06) | 1.12 (1.02, 1.22) | 1.00 (0.96, 1.05) | 1.02 (0.98, 1.06) | 1.10 (1.05, 1.16) ** |
| | Water source | | | | | | |
| | Improved | Reference | Reference | Reference | Reference | Reference | Reference |
| | Unimproved | 0.96 (0.90, 1.02) | 1.08 (1.01, 1.15) | 1.04 (0.97, 1.12) | 1.04 (0.98, 1.09) | 0.97 (0.92, 1.01) | 1.02 (0.97, 1.08) |
| | Child diarrhea | | | | | | |
| | No | Reference | Reference | Reference | Reference | Reference | Reference |
| | Yes | 1.25 (1.15, 1.36) *** | 1.17 (1.09, 1.26) ** | 1.47 (1.35, 1.59) *** | 1.26 (1.19, 1.35) *** | 1.09 (1.03, 1.15) | 1.32 (1.24, 1.41) *** |
| | Child fever | | | | | | |
| | No | Reference | Reference | Reference | Reference | Reference | Reference |
| | Yes | 1.01 (0.95, 1.08) | 1.04 (0.98, 1.11) | 1.10 (1.03, 1.18) | 1.02 (0.97, 1.07) | 0.99 (0.95, 1.04) | 0.99 (0.94, 1.05) |
| | Child cough | | | | | | |
| | No | Reference | Reference | Reference | Reference | Reference | Reference |
| | Yes | 1.04 (0.98, 1.12) | 1.11 (1.05, 1.18) * | 1.08 (1.01, 1.16) | 1.01 (0.96, 1.07) | 1.08 (1.03, 1.12) * | 1.06 (1.01, 1.12) |
| Early learning | Maternal stimulation | | | | | | |
| | Adequate | Reference | Reference | Reference | Reference | Reference | Reference |
| | Inadequate | 1.06 (0.99, 1.14) | 1.17 (1.09, 1.26) *** | 1.23 (1.13, 1.34) *** | 1.03 (0.98, 1.09) | 0.92 (0.88, 0.96) ** | 0.85 (0.80, 0.90) *** |
| | Child books at home | | | | | | |
| | Yes | Reference | Reference | Reference | Reference | Reference | Reference |
| | No | 1.20 (1.04, 1.38) | 1.08 (0.97, 1.21) | 1.17 (0.99, 1.37) | 1.32 (1.24, 1.40) *** | 1.21 (1.16, 1.27) *** | 1.38 (1.29, 1.47) *** |
| | Toys at home | | | | | | |
| | Yes | Reference | Reference | Reference | Reference | Reference | Reference |
| | No | 1.04 (0.95, 1.14) | 1.26 (1.16, 1.38) *** | 1.34 (1.22, 1.48) *** | 0.96 (0.89, 1.03) | 1.34 (1.26, 1.42) *** | 1.45 (1.36, 1.56) *** |
| | ECE attendance | | | | | | |
| | Yes | Reference | Reference | Reference | Reference | Reference | Reference |
| | No | 1.45 (1.32, 1.59) *** | 1.20 (1.11, 1.30) *** | 2.07 (1.85, 2.32) *** | 1.91 (1.81, 2.02) *** | 1.08 (1.03, 1.13) * | 2.27 (2.13, 2.41) *** |
| Safety and security | Birth registration | | | | | | |
| | Yes | Reference | Reference | Reference | Reference | Reference | Reference |
| | No | 1.06 (0.99, 1.13) | 1.14 (1.07, 1.21) ** | 1.24 (1.16, 1.33) *** | 1.23 (1.18, 1.29) *** | 1.03 (0.99, 1.08) | 1.30 (1.23, 1.37) *** |
| | Child supervision | | | | | | |

© 2025 Jeong J et al. *JAMA Network Open.*

| | | | | | | | |
|---|---|---|---|---|---|---|---|
| | Adequate | Reference | Reference | Reference | Reference | Reference | Reference |
| | Inadequate | 1.03 (0.97, 1.09) | 1.11 (1.05, 1.17) ** | 1.12 (1.05, 1.19) * | 1.06 (1.01, 1.11) | 1.19 (1.14, 1.24) *** | 1.23 (1.17, 1.29) *** |
| Enabling environment | Maternal education | | | | | | |
| | Secondary to higher | Reference | Reference | Reference | Reference | Reference | Reference |
| | No or primary | 1.33 (1.23, 1.44) *** | 1.11 (1.04, 1.19) | 1.43 (1.30, 1.56) *** | 1.35 (1.28, 1.42) *** | 1.08 (1.04, 1.12) * | 1.43 (1.35, 1.51) *** |
| | Maternal age | | | | | | |
| | 25-49 years | Reference | Reference | Reference | Reference | Reference | Reference |
| | 15-24 years | 1.17 (1.10, 1.24) *** | 1.01 (0.95, 1.07) | 1.13 (1.06, 1.21) * | 1.18 (1.12, 1.24) *** | 0.98 (0.94, 1.03) | 1.20 (1.14, 1.27) *** |
| | Marital status | | | | | | |
| | Married/ living with a partner | Reference | Reference | Reference | Reference | Reference | Reference |
| | Not married | 1.01 (0.92, 1.10) | 1.00 (0.92, 1.08) | 1.02 (0.94, 1.12) | 0.96 (0.89, 1.05) | 1.08 (1.01, 1.15) | 1.02 (0.94, 1.12) |
| | Household wealth quintile | | | | | | |
| | Richest | Reference | Reference | Reference | Reference | Reference | Reference |
| | Richer | 1.37 (1.22, 1.54) *** | 1.01 (0.92, 1.11) | 1.45 (1.27, 1.65) *** | 1.28 (1.18, 1.38) *** | 1.11 (1.05, 1.17) * | 1.54 (1.41, 1.68) *** |
| | Middle | 1.83 (1.62, 2.07) *** | 1.03 (0.93, 1.15) | 1.71 (1.50, 1.95) *** | 1.50 (1.38, 1.62) *** | 1.26 (1.19, 1.34) *** | 1.99 (1.81, 2.19) *** |
| | Poorer | 1.86 (1.65, 2.10) *** | 1.09 (0.98, 1.22) | 1.90 (1.65, 2.18) *** | 1.88 (1.72, 2.05) *** | 1.25 (1.17, 1.34) *** | 2.51 (2.27, 2.78) *** |
| | Poorest | 2.04 (1.80, 2.31) *** | 1.19 (1.06, 1.33) | 2.13 (1.86, 2.45) *** | 2.25 (2.06, 2.46) *** | 1.39 (1.30, 1.50) *** | 3.07 (2.77, 3.41) *** |
| | Number of children | | | | | | |
| | 0-2 | Reference | Reference | Reference | Reference | Reference | Reference |
| | 3+ | 1.04 (0.97, 1.11) | 1.06 (1.00, 1.12) | 1.10 (1.03, 1.19) | 1.08 (1.03, 1.13) | 1.10 (1.05, 1.15) ** | 1.09 (1.04, 1.15) |
| | Residence | | | | | | |
| | Urban | Reference | Reference | Reference | Reference | Reference | Reference |
| | Rural | 1.32 (1.21, 1.43) *** | 0.98 (0.90, 1.06) | 1.22 (1.11, 1.35) *** | 0.95 (0.90, 1.00) | 0.99 (0.95, 1.04) | 0.91 (0.85, 0.97) |

Note. Regression models included child's age and sex, country and survey year fixed effects, and 17 correlates simultaneously. Clustered standard error was adjusted for. "Neither stunted nor off-track ECD" served as a reference category. P-value is adjusted for Bonferroni correction and signified with * at p<0.05; ** at p<0.01; *** at p<0.001. AOR = adjusted odds ratio. CI = confidence interval. ECD = early childhood development. ECE = early childhood education.

© 2025 Jeong J et al. *JAMA Network Open.*
