# Supplementary material for: Co-Occurrence of Stunting and Off-Track Early Child Development in Low- and Middle-Income Countries
Source: JAMA Netw Open. 2025 Mar 4;8(3):e2462263. doi: 10.1001/jamanetworkopen.2024.62263 (PMC11880945; doi:10.1001/jamanetworkopen.2024.62263)
Supplement: Supplement 2. — Data Sharing Statement [file jamanetwopen-e2462263-s002.pdf]

Data Sharing Statement

Jeong. Co-Occurrence of Stunting and Off-Track Early Child Development in Low- and Middle-Income Countries. *JAMA Netw Open*. Published March 04, 2025.
doi:10.1001/jamanetworkopen.2024.62263

Data
Data available: No
